# Reply from George A. Brooks

**DOI:** 10.1113/JP283189

**Published:** 2022-05-10

**Authors:** George A. Brooks

**Affiliations:** ^1^ Department of Integrative Biology University of California Berkeley CA USA

**Keywords:** cardiac muscle, gluconeogenesis, lactate and brain, lactate shuttle, lactate signalling, lactylation, olfr78, oxidative metabolism

Langan and Navarro are thanked for providing commentary on our paper, ‘Lactate in contemporary biology: a phoenix risen’ (Brooks et al., 2022). We regard their thoughtful letter as a note in addition to our review on the lactate shuttle that is directly related to their provocative title: ‘How high can the lactate phoenix rise?’ (Langan & Navarro, 2022). Clearly, we do not know, but it is imperative to find the apogee.

Regarding the diverse signalling roles of lactate Langan and Navarro noted the role of lactate in activating the carotid body olfactory receptor (Olfr78) (Chang et al., 2015). Give the importance of breathing in physiology, ours was a significant omission making their contribution noteworthy. ‘How high’ indeed!

Langan and Navarro distinguished between the roles of lactate and hypoxia in hypoxia‐inducible factor (HIF) signalling. We touched on that also in our review, but for us to have emphasized the role of lactate over hypoxia in HIF signalling might have been viewed as over reaching.

In our review we noted infancy of the field of histone lactylation on gene expression (Zhang et al., 2019). Already we see extensive evidence not only of histone, but protein lactylation (Leija et al., unpublished). Importantly, the role of lactate as a transcription factor was foreshadowed in an earlier report (Hashimoto et al., 2007).

In our review we briefly touched on the role of lactate as fuelling the spiral mitochondrial reticulum in sperm tails. Most recently, Sharpley et al. commented extensively on the role of lactate in embryogenesis (Sharpley et al., 2020). In terms of a launchpad for studies of lactate's role in biology, what could be more fundamental than the moment of conception and subsequent embryogenesis?

In sum, we thank Langan and Navarro for extending the discussion on the biological roles of lactate, particularly as a signalling molecule. As well, others have made similar observations, for instance the role of lactate in glutamatergic signalling in the brain (Pellerin & Magistretti, 1994). One significant correction we have to offer is to their penultimate sentence and citation on lactate as the primary circulating energy source. The paper cited was confirmatory; there having been many previous primary sources and reviews on lactate as an energy source (e.g. Brooks, 1985; Brooks, 2002).

## Additional information

### Competing interests

None.

### Author contributions

Sole author.

### Funding

NIH: R01 AG059715.

## Supporting information




Peer Review History
